# Exploring the Role of CD44 in the Progression and Invasion of Chondrosarcoma

**DOI:** 10.32604/or.2026.075617

**Published:** 2026-06-16

**Authors:** Zoe Bell, Corey D. Chan, Rachel Howarth, Andrea Atkinson, Zakareya Gamie, Daniel Frankel, Oana Bretcanu, Kenneth S. Rankin

**Affiliations:** 1 Newcastle Centre for Cancer, Newcastle University, Paul O’Gorman Building, Framlington Place, Newcastle upon Tyne, UK; 2 North of England Bone and Soft Tissue Tumour Service, Freeman Hospital, Newcastle upon Tyne Hospitals NHS Foundation Trust, Newcastle upon Tyne, UK; 3 Translational and Clinical Research Institute, Newcastle University, Newcastle upon Tyne, UK; 4 Orthopaedic Oncology, Newcastle upon Tyne Hospitals NHS Foundation Trust, Newcastle upon Tyne, UK; 5 School of Engineering, Newcastle University, Newcastle upon Tyne, UK

**Keywords:** Chondrosarcoma, cluster of differentiation 44 solid tumour, immunohistochemistry, clustered regularly interspaced short palindromic repeats, spheroid, invasion

## Abstract

**Objectives:** Chondrosarcoma is the most common type of primary bone sarcoma in adults with a high risk of local recurrence and metastasis. Chondrosarcomas are largely resistant to chemotherapy and radiotherapy, meaning that surgery is the mainstay of treatment for most patients. Therefore, new therapeutic targets are required. Cluster of differentiation 44 (CD44) is a transmembrane protein that has roles in cell proliferation, adhesion and migration and is shown to be overexpressed in several cancer types. Consequently, this work was undertaken to understand whether CD44 could be a potential therapeutic target in chondrosarcoma. **Methods:** In this study, tissue sections from chondrosarcoma patients were evaluated for CD44 expression using immunohistochemical analysis. The relationship between CD44 expression in the patient tissue and overall and event-free survival was investigated. Clustered regularly interspaced short palindromic repeats (CRISPR)/CRISPR-associated protein 9 (Cas9) gene editing was used to generate a CD44 knockout chondrosarcoma cell line which was used for a spheroid invasion assay to understand the role of CD44 in chondrosarcoma cell invasion. **Results:** Cox multivariate analysis of CD44 expression in chondrosarcoma patient tissue revealed that high CD44 expression was linked to decreased overall (*p* < 0.01) and event-free survival (*p* < 0.01). The observed invasion of CD44 knockout cells in the spheroid assay was less than that of the wild type cells (*p* < 0.001). **Conclusions:** The increased expression of CD44 in intermediate and high grade chondrosarcomas, as well as reduced invasion of CD44 knockout cells suggests that CD44 plays an important role in chondrosarcoma progression and metastasis. These findings support further investigation of CD44 as an imaging and/or therapeutic target for the management of chondrosarcoma.

## 1 Introduction

Chondrosarcoma is the most common type of primary bone sarcoma in adults and is characterised by the production of a cartilaginous matrix [1]. Chondrosarcoma tumours most often arise in the pelvis and long bones such as the femur and humerus [2]. Between 1998 and 2007, an average of 113 patients were diagnosed with chondrosarcoma each year in England [3]. The most common age range for chondrosarcoma to occur is 50–70 years old and it has been shown that younger patients with chondrosarcoma generally have a more favourable prognosis [4]. The 2020 World Health Organization classification of chondrosarcomas states the following subgroups: conventional central (grades 1, 2 and 3), secondary peripheral (grades 1, 2 and 3), periosteal, dedifferentiated, mesenchymal, and clear cell chondrosarcoma. Grade 1 chondrosarcomas, also termed atypical cartilaginous tumours (ACTs), are characterised by the presence of chondrocytes with small nuclei, whereas characteristics of a grade 2 tumour include less matrix and more cells with enlarged nuclei [1]. Grade 3 tumours are the most invasive, are more cellular with less matrix and have large, often spindle-shaped, nuclei [5]. Dedifferentiated chondrosarcomas are identified by a low-grade cartilaginous component which abruptly transitions to a high-grade non-cartilaginous component. Patients with dedifferentiated chondrosarcoma have a very poor prognosis due to its highly invasive nature which often results in widespread metastatic disease [6]. Dedifferentiated chondrosarcomas are sometimes referred to as grade 4 tumours, and have a poor 5-year overall survival estimate of 32% [7]. Metastasis occurs in approximately 24% of chondrosarcoma patients with the risk of metastasis increasing with tumour grade [8].

The mainstay of treatment for chondrosarcoma is wide surgical resection. For high grade chondrosarcoma, a clear surgical margin of at least 4 mm is recommended to reduce the risk of local recurrence [9]. However, a sufficient tumour margin is not always possible, especially in the pelvis where critical neurovascular structures are in very close proximity to, or even involve, the tumour [10]. Chondrosarcomas are largely resistant to adjuvant therapies but, certain conventional chemotherapy agents such as doxorubicin or cisplatin can show some effect in mesenchymal and dedifferentiated chondrosarcomas [11]. Additionally, apart from tumours arising in the skull base, chondrosarcomas are resistant to radiotherapy and therefore it is only considered for patients where surgery is not possible, or those with incomplete resection [12]. Although low grade chondrosarcomas can be treated by surgery, the extent of local tumour invasion and the risk of metastasis is much lower than with higher grade disease [13]. This, combined with the widespread resistance of chondrosarcomas to chemotherapy and radiotherapy highlights the urgent need for new therapeutic strategies, especially for those patients with metastatic or inoperable disease.

Cluster of differentiation 44 (CD44) is a transmembrane glycoprotein expressed by lymphocytes, smooth muscle cells, fibroblasts, and epithelial cells. In healthy tissues CD44 contributes to cell adhesion, aggregation and cytokine release [14]. CD44 consists of 19 exons in humans where the first and last five exons are the same for all forms of CD44 and it is these 10 exons that form the standard form of CD44 (CD44s). Up to nine of the remaining central exons are alternatively spliced to form the variant forms of CD44 (CD44v2–CD44v10) [15].

CD44 is the main receptor of hyaluronic acid but other ligands of CD44 are collagen, fibronectin and laminin [16]. When hyaluronic acid binds to CD44 on the cell surface, the interaction of hyaluronic acid with CD44 can regulate pathways that are involved in cell proliferation and migration as well as angiogenesis and inflammation [17]. For example, hyaluronic acid binding to CD44 has been shown to activate the phosphatidylinositol 3 kinase/AKT pathway which is important in cell cycle regulation. This occurs because rho-associated coiled coil containing protein kinase (ROCK) activity is stimulated by hyaluronic acid binding to CD44. This then leads to an increase in serine/threonine phosphorylation of the GRB2-associated binding protein 1 which subsequently activates phosphatidylinositol 3 kinase and this activates AKT signalling [18]. AKT promotes cell proliferation and survival by inactivating pro-apoptotic proteins [19]. Binding of hyaluronic acid to CD44 can also promote cell migration via actin cytoskeleton contractility. Through activating the Rho guanosine triphosphatase Ras homolog family member A (RhoA) to activate ROCK, it signals the phosphorylation of myosin which is required for cell migration [20].

Increased CD44 expression correlates with tumour grade for a range of cancer types including ovarian [21], breast cancer [22], prostate adenocarcinoma [23] and meningioma [24]. In carcinomas, CD44 may contribute to cancer cell invasion due to its role in the process of epithelial-mesenchymal transition (EMT). During EMT, epithelial cells transition to invasive mesenchymal cells through morphogenic changes and altered cell surface expression [25]. It has been shown that during EMT, CD44 is required to change isoforms from CD44v to CD44s. CD44s can activate AKT resulting in a reduction in epithelial cadherin expression which is needed for cell-cell adhesion in epithelial cells. Consistent with this, CD44 knockdown resulted in inhibition of EMT due to the unavailability of CD44s [26]. Additionally, CD44 has been shown to lead to an increase in angiogenesis. Angiogenesis is an important process in tumour growth and invasion because neovasculature can provide the tumour with nutrients and oxygen as well as the removal of waste products which prevents the tumour from becoming necrotic [27]. The presence of CD44 on blood vessel walls allows endothelial cells to adhere to extracellular matrix (ECM) components which are required for the formation of blood vessels. Hyaluronic acid binding to CD44 can also lead to an increase in vascular endothelial growth factor expression which is required for the formation of new blood vessels [28]. CD44 can also enhance cancer cell invasion by promoting the secretion of matrix metalloproteinase-9 (MMP-9), which degrades type IV collagen—the predominant collagen component in the basement membrane of the ECM [29]. Furthermore, as CD44 can act on the cell surface membrane, it can form clusters which retain MMP-9 on the cell membrane, promoting membrane degradation and enabling tumour cell invasion [30]. Additionally, CD44 on the cell surface facilitates the recycling of membrane type 1 MMP which allows cells to maintain degradation of the ECM which is an essential step in cancer cell invasion [31].

One of the objectives of this study was to investigate whether CD44 could be used as a future therapeutic or imaging target for chondrosarcoma. A clinical trial of RG7356 (ID NCT01358903), a recombinant immunoglobin G1 monoclonal antibody which binds near the hyaluronic acid binding region of CD44 has been undertaken on patients with CD44-expressing solid tumours, although no patients had chondrosarcoma [32]. The results of the trial showed that although no safety concerns with RG7356 arose, the clinical efficacy was modest. A subgroup of the patients involved in this trial were also recruited for imaging studies using positron emission tomography (PET) imaging with 89Zirconium-labelled RG7356 [33]. The PET imaging revealed that tumour uptake of the CD44 antibody in some tissues was dose-dependent suggesting that immune-PET imaging with this antibody could be beneficial for a non-invasive measure of drug uptake.

In this study, CD44 expression in chondrosarcoma patient tissue and patient-derived chondrosarcoma cells was characterised to evaluate whether CD44 could be a potential therapeutic target for chondrosarcoma. Previously, Heyse et al. undertook a similar study which involved staining tissue samples from 30 chondrosarcoma patients using antibodies for CD44s, CD44v5 and CD44v6 [34]. Survival analysis revealed that higher CD44s expression contributed to decreased overall survival (OS) and metastatic free survival. Here, previous work is expanded upon by utilizing a larger patient cohort to increase the understanding of CD44 expression and to examine the prognostic value of CD44. The potential of CD44 as a therapeutic target for chondrosarcoma has been investigated by Yoshida et al. [35]. SW 1353 chondrosarcoma cells were treated with the anti-CD44 monoclonal antibody, IM7, and it was shown that IM7 decreased viability of the SW 1353 cells in a dose-dependent manner. Therefore, in our study, one of the main objectives was to further elucidate the role of CD44 in chondrosarcoma cell invasion, given that it is a key process in the progression of chondrosarcoma and represents a critical area of investigation for potential treatment modalities. For the first time in this work, clustered regularly interspaced short palindromic repeats/CRISPR associated protein 9 (CRISPR/Cas9) gene editing was used to knockout CD44 in the HT-1080 chondrosarcoma cell line which were subsequently used in a spheroid invasion assay to directly evaluate the role of CD44 in chondrosarcoma cell invasion. In summary, the aim of this study was to evaluate CD44 expression in a large patient cohort and, assess CD44 function in commercially available cells as well as cells derived from patients recently treated at our centre with the hypothesis that CD44 is important for promoting chondrosarcoma invasion and tumour progression.

## 2 Materials and Methods

### 2.1 Cell Culture

The HT-1080 human dedifferentiated chondrosarcoma cell line was purchased from American Type Culture Collection (CCL-121, Manassas, VA, USA) and authenticated by northgene (Deeside, UK) using short tandem repeats analysis. The HT-1080 cell line was originally reported as a ‘bone fibrosarcoma’ as a diagnosis of exclusion. However, it is now known to harbour an isocitrate dehydrogenase 1 (IDH1) mutation which the current literature suggests is most representative of an aggressive dedifferentiated chondrosarcoma [36,37]. The patient-derived cells were extracted from the patient tissue as previously outlined [38,39]. Both the HT-1080 and patient-derived cells were tested for the presence of mycoplasma. Patient-derived cells from two different patients were cultured. The 13095 cells were derived from the pelvis of a patient with a grade 2 secondary chondrosarcoma arising from an osteochondroma. The 13073 cells were derived from the pelvis of a patient with a grade 3 primary chondrosarcoma. These patient-derived cell lines were chosen because they were derived from patients with different chondrosarcoma grades and anatomical sites. These cells were also found to be robust in culture. Both the HT-1080 cells and patient-derived cells were cultured in Roswell Park Memorial Institute 1460 medium (Sigma-Aldrich, R8758, St. Louis, MO, USA) supplemented with 10% foetal bovine serum (Gibco, A5256701, Waltham, MA, USA) and 1% penicillin-streptomycin (Sigma-Aldrich, P0781, St. Louis, MO, USA). All cells were cultured in a humidified incubator at 5% CO_2_ and 37°C in a T-25 or T-75 cell culture flask (Corning, 430639 and 430641U, New York, NY, USA). The cells were subcultured when they reached a confluency of 80–95%.

### 2.2 Immunohistochemistry

#### 2.2.1 Patient Tissue Slides

Chondrosarcoma tissue from 46 patients was obtained from the Newcastle Biobank (IRAS 233551, REC 17/NE/0361). The tissues were collected between 2011 and 2023. The study was conducted according to the guidelines of the Declaration of Helsinki and approved by the Institutional Review Board of Newcastle upon Tyne Hospitals NHS Hospitals Trust (Caldicott number 7159, 10 June 2019). Written informed consent for their tumour tissues to be used in sarcoma research was obtained from all subjects involved in the study. For minors, an assent form was used and signed by their parent/legal guardian. A total of 49 tissue sections were included in the analysis as two patients had multiple slides due to recurrence. The tumours were confirmed as chondrosarcoma and graded by specialist sarcoma pathologists at the North of England Bone and Soft Tissue Tumour Service. Tissues were obtained from patients with a median age of 54 (range 10–89 years). A total of 20 tissue samples were obtained from female patients and 29 samples were obtained from male patients. The tissue was sliced into 4 μm slices. The sections were taken from chondrosarcomas with different grades including 14 grade 1/ACT, 17 grade 2, 18 grade 3.

#### 2.2.2 Generation of Patient-Derived Cell Block

Cell blocks for the 13095 and 13073 patient-derived cells were created by trypsinising two confluent T-75 flasks for each cell line and centrifuging them at 250× *g* for 5 min. The cell pellet was washed in phosphate-buffered saline (PBS) and centrifuged again. The cell pellet was resuspended in 2 mL 4% formaldehyde (Genta Medical, BFN010, York, UK) for 1 h. The cells were centrifuged at 250× *g* for 5 min and resuspended in 300 μL HistoGel (Epredia, HG-4000-012, Portsmouth, NH, USA) and then centrifuged for 30 s at 16,500× *g*. The cells were then placed on ice for 30 min. The cells in the HistoGel were transferred into a tissue cassette (Simport, M492, Beloeil, Quebec, Canada) and then processed using a Leica (Wetzlar, Germany) TP 1020 tissue processor. Processing steps included placing the samples in ethanol (Thermo Fisher Scientific, E/065DF/17, Waltham, MA, USA), xylene (VWR, 28975.325 Radnor, PA, USA) and paraffin wax (CellPath, cellwax plus GCA-0400-00A, Newtown, UK). Finally, the cell pellet was encased in paraffin wax using a modular tissue embedding centre (Myr, EC 350, Tarragona, Spain) and once set, it was sliced into 4 μm slices using a microtome (Leica RM2135, Wetzlar, Germany). The slices were placed on slides (VWR, 631-0108, Radnor, PA, USA) and heated for 2 h to dry in preparation for staining.

#### 2.2.3 Antibody Staining of Patient Tissue and Cell Block Slices

The slides were first de-waxed and hydrated. Initially, the slides were soaked in three separate tubs of xylene (VWR, 28975.325, Radnor, PA, USA) for 5 min each time. They were then soaked in three separate tubs of 100% ethanol (Thermo Fisher Scientific, E/065DF/17, Waltham, MA, USA) for 1 min each time. This was followed by 95% ethanol for 1 min, 70% ethanol for 1 min, then 50% ethanol for 1 min and then distilled water for 1 min. Antigen retrieval was carried out by placing the slides in a pressure cooker containing citrate buffer. The citrate buffer consisted of 2.1 g citric acid (Thermo Fisher Scientific, 423565000, Waltham, MA, USA) dissolved in 750 mL distilled water with 2 M sodium hydroxide (Scientific Laboratory Supplies, 71474, Nottingham, UK) added until a pH of 6 was reached. Distilled water was added to make the solution up to 1 L. The slides were heated in the pressure cooker for 15 min before turning the pressure cooker off and allowing it to cool. The slides were then washed in tris-buffered saline (TBS) on a rocker for 5 min, three times. The TBS solution was made from diluting 500 mL 20× stock in 9.5 l distilled water. The 20× stock was made by dissolving 26 g tris(hydroxymethyl)aminomethane (Sigma-Aldrich, 252859, St. Louis, MO, USA) and 320 g sodium chloride (Acros organics, 207790050, Geel, Belgium) in 15 mL 6 M hydrochloric acid solution (Honeywell, 017-002-01-X, Charlotte, NC, USA). The pH was then adjusted to 7.6 using 1 M hydrochloric acid solution (Honeywell, 017-002-01-X, Charlotte, NC, USA) and then the volume was made up to 2 L using distilled water. During the wash, a 3% hydrogen peroxide solution was made by diluting 30% stock hydrogen peroxide (Thermo Fisher Scientific, H/1750/15, Waltham, MA, USA) 1:10 in TBS. The slides were then washed in the hydrogen peroxide solution for 15 min on a rocker. Next, the slides were washed in TBS-T for 5 min on a rocker three times. TBS-T consisted of 9480 mL distilled water, 20 mL Tween 20 (Promega, H5151, Madison, WI, USA) and 500 mL 20× TBS. During this time, the blocking solution was made which consisted of 5% goat serum (Capricorn Scientific, GOA-1B, Ebsdorfergrund, Germany) in 1% bovine serum albumin (BSA, Sigma-Aldrich, 810033, St. Louis, MO, USA) in TBS. The excess liquid was then tapped off a slide and a hydrophobic pen (Thermo Fisher Scientific, R3777, Waltham, MA, USA) was used to draw around the area of cells or tissue that needed to be stained. 100 μL of the blocking solution was added within the area drawn by the hydrophobic pen and the slide was left in a humidity chamber at room temperature for 1 h. The blocking solution was removed from each slide and 100 μL of the primary antibody solution was added to each slide which consisted of the CD44 antibody (monoclonal, mouse anti-human, Cell Signalling, 5640S, Danvers, MA, USA) diluted 1:1000 in TBS for the cell block slices and 1:500 for the patient tissue. This CD44 antibody detects all isoforms of CD44 except isoforms 2, 9, 15 and 19. The slides were left in the humidity chamber overnight at 4°C. The following day, the primary antibody solution was removed from the slides, and they were washed in TBS-T for 5 min three times. During the washes, the secondary antibody solution was made which consisted of polyclonal goat anti-mouse immunoglobulins conjugated with horseradish peroxidase (HRP, Dako, P044701-2, Santa Clara, CA, USA) diluted 1:1000 in TBS. The secondary antibody was left on the slides for 1 h at room temperature in a humidity chamber. The secondary antibody solution was removed from the slides and they were washed in TBS-T for 5 min three times. The 3,3′-diaminobenzidine (DAB) solution was made by mixing 30 μL SignalStain DAB Chromogen Concentrate with 1 mL SignalStain DAB Diluent (Cell Signalling, 8059, Danvers, MA, USA). 100 μL of the DAB solution was added to each slide for 5 min. The DAB solution was removed from each slide and the slides were washed in DI water for 3 min. The slides were then rapidly dipped in Gill 3 hematoxylin (Epredia, 6765009, Portsmouth, NH, USA) to counterstain the slides. Once dipped, the slides were washed in running tap water for 3 min, placed in Scott’s tap water for 1 min and washed in running tap water again for 3 min. Scott’s tap water consists of 2 L of distilled water, 7 g sodium hydrogen carbonate (Thermo Fisher Scientific, A17005.36, Waltham, MA, USA) and 40 g magnesium sulfate heptahydrate (Thermo Fisher Scientific, A14491, Waltham, MA, USA). The slides were then dehydrated by placing them in 50% ethanol (Thermo Fisher Scientific, E/065DF/17, Waltham, MA, USA) for 1 min, 95% ethanol for 1 min, 70% ethanol for 1 min, 50% ethanol for 1 min then 2 min in three separate containers of xylene (VWR, 28975.325, Radnor, PA, USA). The slides were then mounted with cover slips using a dibutylphtalate polystyrene xylene mountant (Sigma-Aldrich, 06522, St. Louis, MO, USA) which was left overnight to set. All slides were scanned using a Leica (Wetzlar, Germany) Aperio CS2 using a 20× objective lens.

#### 2.2.4 Quantification of Antibody Staining

QuPath software (version 0.5.1, Edinburgh, UK) was used to quantify the staining intensity of the patient tissue slides that had been stained with an antibody for CD44 [40]. The CD44 staining on the chondrosarcoma patient tissue slices was quantified using the positive cell detection tool on QuPath. An area of interest on the tissue was first selected using the annotation tool before running positive cell detection which resulted in the histochemical score (H-score) of the region of interest. The H-score is calculated by: (1 × percentage of weak staining) + (2 × percentage of moderate staining) + (3 × percentage of strong staining), resulting in a value between 0 and 300 [41]. A summary of the CD44 antibody that was used and QuPath staining thresholds is summarised in Table 1.

**Table 1:** This table contains information about the CD44 antibody used for the immunostaining of the cell block slices and chondrosarcoma patient tissue slices. The threshold values were used in QuPath software when quantifying the staining and determining the H-score.

Antibody Type	Mouse Monoclonal
Dilution–Cell Block	1:1000
Dilution–Patient Tissue	1:500
QuPath Threshold: Weak	0.05
Moderate	0.1
High	0.2

### 2.3 Flow Cytometry

Cells were washed with PBS then incubated for up to 20 min at 37°C, and 5% CO_2_ with 10 mL flow buffer consisting of 50 mL PBS, 2.5 mL 0.2 mM ethylenediaminetetraacetic acid (Sigma-Aldrich, E6758 St. Louis, MO, USA) and 2.5 mL MACS BSA (Miltenyi Biotec, 130-091-376, Bergisch Gladbach, Germany). 1 × 10^6^ cells in 100 μL flow buffer were transferred to FACS tubes (Falcon, Corning, 352003, New York, NY, USA) on ice. An additional 650 μL flow buffer was added to the tube that would be the unstained sample. For the stained samples, 3 μL human Seroblock (Bio-Rad, BUF070A, Hercules, CA, USA) was added to each tube and incubated on ice for 10 min. Then either 3 μL fluorescein isothiocyanate (FITC) isotype control antibody (BD Biosciences, 555748, Franklin Lakes, NJ, USA) per 1 × 10^6^ cells or 3 μL CD44 FITC antibody (monoclonal, mouse anti-human, BD Biosciences, 560977, Franklin Lakes, NJ, USA) per 1 × 10^6^ cells was added to the relevant tubes and incubated on ice in the dark for 50 min. The cells were then washed twice by adding 1 mL flow buffer to the tubes followed by centrifugation at 250× *g* for 5 min. The pellets were resuspended in 750 μL flow buffer and the samples were analysed using a BD LSRFortessa X-20 (BD Biosciences, Franklin Lakes, NJ, USA) with the 488 nm laser and 530/30 bandpass filter. Dead cells and doublets were excluded by the use of appropriate gating. Flow cytometry data was analysed using FCS Express 7 software (*De Novo* software, Pasadena, CA, USA).

### 2.4 CD44 Knockout

#### 2.4.1 Lipofectamine CRISPR Transfection

The day prior to the transfection, 1 × 10^5^ cells were seeded in the wells of a 24 well plate (Corning, 3524, New York, NY, USA). On the day of the transfection, the old media was removed and replaced with fresh media. The TrueGuide Synthetic single guide RNA (sgRNA) (Thermo Fisher Scientific, CRISPR807011_SGM, Waltham, MA, USA) was diluted in Opti-MEM (Thermo Fisher Scientific, 31985062, Waltham, MA, USA) to a concentration of 250 ng/μL. In a 1.5 mL microcentrifuge tube, 8.25 μL Lipofectamine MessengerMax (Invitrogen, LMRNA015, Waltham, MA, USA) and 137.5 μL Opti-MEM were vortexed and incubated at room temperature for 5 min. In a different 1.5 mL microcentrifuge tube, 50 μL Opti-MEM and 1 μL GeneArt Cas9 Nuclease 1 μg/μL (Thermo Fisher Scientific, A29378, Waltham, MA, USA) were mixed well by pipetting. 1 μL of the 250 ng/μL sgRNA was also added to this tube which was then mixed well by pipetting followed by vortexing. 50 μL of the diluted Lipofectamine MessengerMax solution from the first tube was then added to the second tube. The solution was mixed by gentle pipetting. The tube was incubated for 15 min at room temperature. 50 μL of this solution was added to the well that contained the cells to be transfected. The plate was gently agitated and incubated at 37°C for 40–48 h. The old media was then removed from the wells and the cells were washed with PBS, trypsinized and transferred to a T-25 (Corning, 430639, New York, NY, USA) flask to be continuously cultured.

#### 2.4.2 Fluorescence Activated Cell Sorting

Following flow cytometry to check that the CRISPR knockout was successful, cells were sorted using fluorescence activated cell sorting (FACS) to separate the wild type (WT) cells from the knockout cells. Cells were prepared for sorting by following the flow cytometry protocol. The media that was aspirated from the flasks containing the cells was kept and sterile filtered using a 0.2 μm filter (Sartorius, 17823, Göttingen, Germany) and a 20 mL syringe (Becton Dickinson, 613-3921, Franklin Lakes, NJ, USA). The filtered media was used to make conditioned media by mixing it with equal quantities of fresh media. Each well of a 96 well plate was filled with 100 μL of the conditioned media prior to the sort. Before sorting, 5 μL DAPI was also added to each tube (Roche, 10236276001, Basel, Switzerland). The cells were sorted using a BD FACSAria Fusion flow cytometer (Becton Dickinson, Franklin Lakes, NJ, USA) using the 100 μm nozzle where single cells were deposited into each well of the 96 well plate (Corning, 35978, New York, NY, USA) containing the conditioned media. Gating was used to discard dead cells, doublets, debris and cells that were bound to the CD44 antibody (monoclonal, mouse anti-human, BD Biosciences, 560977, Franklin Lakes, NJ, USA) meaning only cells that had CD44 knocked out were deposited into the plate. The single cells were then continuously cultured.

### 2.5 Western Blot

Cells were lysed using a cell scraper (Thermo Fisher Scientific, 11577692, Waltham, MA, USA) in RIPA solution consisting of 490 μL Pierce RIPA buffer (Thermo Fisher Scientific, 89900, Waltham, MA, USA) 5 μL Halt protease inhibitor single use cocktail 100× and 5 μL 0.5 M ethylenediaminetetraacetic acid solution 100× (Thermo Fisher Scientific, 78430, Waltham, MA, USA). Lysates were obtained following centrifugation at 16,500× *g* for 10 min at 4°C. The protein present in the lysates was quantified using the Pierce bicinchoninic acid protein assay kit (Thermo Fisher Scientific, 23225, Waltham, MA, USA) according to the manufacturer’s instructions. Sodium dodecyl sulfate polyacrylamide gel electrophoresis was then undertaken as follows. 40 μg of the protein samples were mixed with RIPA buffer to reach a total volume of 26 μL. 4 μL of BOLT reducing agent (Thermo Fisher Scientific, B0009, Waltham, MA, USA) and 10 μL of BOLT sample buffer (Thermo Fisher Scientific, B0007, Waltham, MA, USA) were also added to each sample which were then boiled at 100°C for 10 min to denature the proteins. Samples were then centrifuged at 16,500× *g* for 30 s at room temperature. The samples were loaded into a pre-cast Bis-Tris gel (Bio-Rad, 3450112, Hercules, CA, USA) alongside 5 μL of a protein ladder (Bio-Rad, 1610375, Hercules, CA, USA). Running buffer comprising of 50 mL 20× BOLT MES SDS running buffer (Thermo Fisher Scientific, B0002, Waltham, MA, USA) and 950 mL DI water was added to the tank (Bio-Rad, 1656001, Hercules, CA, USA) and the gel was ran at 100 V for 2 h. The proteins were transferred to nitrocellulose membranes (Cytiva, 10600007, Marlborough, MA, USA) which was blocked using a 5% milk/TBS-T solution for 1 h. The membrane was then incubated with either a 1:1000 dilution of the CD44 antibody (Cell Signalling, 5640S, Danvers, MA, USA) or a 1:5000 dilution of the glyceralydehyde-3-phosphate dehydrogenase (GAPDH) control antibody (Cell Signalling, 2118, Danvers, MA, USA) overnight at 4°C. Following this, the membrane was washed five times with TBS-T for five min at room temperature. A 1:1000 dilution of goat anti-rabbit secondary antibody (DAKO, P044801-2, Santa Clara, CA, USA) was then added to the membrane for 1 h at room temperature. Following four washes in TBS-T and one wash of TBS, the membrane was visualised by adding 500 μL of enhanced chemiluminescence solution (Bio-Rad, 1705061 Hercules, CA, USA) to the membrane for 5 min at room temperature. The membrane was then imaged using a Bio-Rad (Hercules, CA, USA) ChemiDoc imaging system.

### 2.6 Quantitative Reverse Transcription Polymerase Chain Reaction (qRT-PCR)

#### 2.6.1 RNA Extraction

Cells for RNA extraction were grown in the wells of a 6 well plate (Corning, 3516, New York, NY, USA) until confluency. The RNA was extracted using the RNeasy mini kit (Qiagen, 74104, Hilden, Germany) as per the manufacturer’s instructions for animal cells.

#### 2.6.2 Complementary DNA Synthesis

The complementary DNA (cDNA) was synthesised from the RNA samples using the Applied Biosystems High-Capacity cDNA Reverse Transcription kit (Thermo Fisher Scientific, 4368814, Waltham, MA, USA) according to the manufacturer’s instructions. The master mix was prepared which consisted of 2 μL 10× RT buffer, 0.8 μL 25× dNTP mix, 2 μL 10× RT random primers, 1 μL MultiScribe reverse transcriptase and 4.2 μL nuclease free water per reaction. 1000 ng of each sample was prepared in 10 μL molecular grade water (waternation, MLG1, Widness, UK) which was mixed with 10 μL of the master mix in a polymerase chain reaction (PCR) tube (Starlab, I1405-8100, Hamburg, Germany) and then centrifuged for 10 s at 16,500× *g*. A Techne Prime thermocycler (Minneapolis, MN, USA) was then used to run the samples in the cycle outlined in the manufacturer’s instructions. Samples were then centrifuged for 10 s at 16,500× *g* and stored at −20°C.

#### 2.6.3 Reverse Transcription-PCR

For each 10 μL reaction, a master mix was made which consisted of 5 μL Power-Track SYBR green (Thermo Fisher Scientific, A46012, Waltham, MA, USA), 2 μL molecular grade water, 0.5 μL forward primer (Thermo Fisher Scientific, 10336022, Waltham, MA, USA) and 0.5 μL reverse primer (Thermo Fisher Scientific, 10336022, Waltham, MA, USA). The primer sequences were chosen so that they were away from any sections of DNA that were knocked out as this would prevent the primers from binding to KO cell lines. The CD44 primer sequences were taken from Zhang et al. and the forward primer sequence was 5′-CAGCTCATACCAGCCATCCA and the reverse was 5′-TGGGGTGTGAGATTGGGTTG [42]. Hypoxanthine phosphoribosyltransferase 1 was used as the housekeeper gene as a reference and the forward sequence used was 5′-TTGCTTTCCTTGGTCAGGCA and the reverse sequence was 5′-AGCTTGCGACCTTGACCATCT. The primers were used at a concentration of 10 μM. To prepare the samples for quantitative reverse transcription PCR (qRT-PCR), 2 μL of cDNA from each sample that had been diluted 1:3 in molecular grade water and 8 μL of the master mix was added to each well of a 384 well plate (Thermo Fisher Scientific, 4343370, Waltham, MA, USA). Each sample was seeded in triplicate. The plate was then sealed with optical adhesive film (Thermo Fisher Scientific, 4360954, Waltham, MA, USA) and briefly centrifuged. The QuantStudio 7 Flex Real-Time PCR system (Thermo Fisher Scientific, 4485701, Waltham, MA, USA) was used to run the qRT-PCR using a standard run for 40 cycles. The results of the qRT-PCR were analysed using QuantStudio Real-Time PCR software (Thermo Fisher Scientific, Waltham, MA, USA).

### 2.7 Sanger Sequencing

#### 2.7.1 DNA Extraction

DNA was extracted from cell pellets using QuickExtract DNA Extraction Solution (Lucigen, QE09050, Middleton, WI, USA) as per the manufacturer’s instructions.

#### 2.7.2 Primer Design

First, the genomic sequence of CD44 (NG_008937.1) was obtained from the National Center for Biotechnology Information (NCBI) website (https://www.ncbi.nlm.nih.gov/nuccore/211904170) [43]. The target DNA sequence from the TrueGuide sgRNA was highlighted on the sequence and 200 base pair (bp) upstream and downstream of the sgRNA sequence was isolated from the full genomic sequence. This was then input into the NCBI Primer-BLAST website to design the primers with an amplicon length of 200–250 bp selected [44]. The generated primer pairs were then checked to ensure that the sgRNA sequence was centrally located between the forward and reverse primers. Once that condition was met, the primers were also checked to ensure the following conditions were also met: the length of each primer was 16–28 bp, the GC content was 40–60%, the melting temperature was 52–62°C and the difference in melting temperature between the forward and reverse primer was less than [45]. The CD44 forward primer sequence that was used was 5′-GGAGTCTGTCCTAAACTGAACTTA and the reverse sequence was 5′-GCAGGTCTCAAATCCGATGC (Eurofins Genomics, 12PP-001PPT, Wolverhampton, UK).

#### 2.7.3 PCR

PCR was used to amplify the required DNA fragments. The forward and reverse primers were first suspended in molecular grade water to achieve a stock concentration of 100 pmol/μL. This was then used to make a working concentration of 10 pmol/μL. For each DNA sample, the following PCR mix was made: 25 μL Premix Taq DNA Polymerase Hot Start Version (TaKaRA, R028A, San Jose, CA, USA), 25 μL molecular grade water, 2 μL DNA extract, 1 μL 10 pmol/μL forward primer and 1 μL 10 pmol/μL reverse primer. The PCR was run using a Techne Prime Thermocycler (Minneapolis, MN, USA) with the lid pre-heated to 105°C and the following cycle: 10 min at 95°C, 40 cycles of 95°C for 30 s, 60°C for 30 s, 72°C for 30 s and then 72°C for 7 min with a hold temperature of 10°C.

#### 2.7.4 PCR Purification

The PCR products were purified using the QIAquick PCR Purification kit (Qiagen, 28104, Hilden, Germany) according to the manufacturer’s instructions for purification using a centrifuge. All centrifugation steps took place at room temperature at 15,000× *g*.

#### 2.7.5 Sanger Sequencing and Analysis

20 μL of each purified PCR product was prepared to a concentration of 1 ng/μL in molecular grade water. The samples and 65 μL of the forward primer at 10 pmol/μL were labelled and sequenced using Eurofins Genomics (Wolverhampton, UK) TubeSeq Supreme service. The .ab1 files that were obtained from Eurofins were then input into the Synthego Inference of CRISPR Edits (ICE) software for analysis [46].

### 2.8 Cell Proliferation Assay

To investigate the proliferation of the HT-1080 wild type (WT) and HT-1080 CD44 knockout (KO) cells, a cell counting kit-8 (CCK-8) cell proliferation assay was used. Cells were seeded in a 96 well microplate (Corning, 3598, New York, NY, USA) at a density of 4000 cells/well in 100 μL of media and allowed to adhere overnight. The following day 10 μL of the CCK-8 solution (Doijndo Laboratories, CK04-13, Kumamoto, Japan) was added to each well and the plate was incubated for 2 h at 37°C. The plate was then read at 450 nm using the FLUOstar Omega Microplate reader (BMG LABTECH, Ortenberg, Germany). Blank wells containing just media and the CCK-8 solution were included for analysis as a control. Following this the wells were washed with PBS and media added. The assay was then repeated after 48 h for a total 72 h incubation period. The percentage increase in optical density following the 72 h incubation period, compared to day one, was then calculated to determine any difference in proliferation rate between the HT-1080 WT and KO cells because of the KO.

### 2.9 Collagen Spheroid Invasion Assay

To generate the spheroids, HT-1080 cells were seeded in the wells of a 96 well ultra-low attachement microplate (Corning, 7007, New York, NY, USA) at a density of 3000 cells/well and incubated at 37°C for 72 h. Once the spheroids had formed, the media was removed from the well containing the spheroid and replaced with 100 μL of either a type I collagen hydrogel or a type I collagen hydrogel with hyaluronic acid. The collagen hydrogel solution was formed as follows: at least 24 h before encasing the spheroids in collagen, 10 mg lyophilised type I collagen from rat tail tendon (Roche, 11179179001, Basel, Switzerland) was dissolved in 3.3 mL 0.2% acetic acid diluted from 100% stock (Sigma-Aldrich, A6283, St. Louis, MO, USA) to achieve a collagen concentration of 3 mg/mL which was stored at 4°C. On the day that the spheroids were encased in collagen, 500 μL 0.33 M sodium hydroxide and 500 μL Dulbecco’s Modified Eagle’s Medium 10× (Sigma-Aldrich, D2429, St. Louis, MO, USA) were vortexed to mix and labelled as solution A, which was stored on ice. For the hydrogels just containing collagen, 500 μL of the collagen solution was mixed with 125 μL of solution A by gentle pipetting to avoid the formation of bubbles in the hydrogel. For the collagen hydrogels with hyaluronic acid, 100 mg medium molecular weight hyaluronic acid (Bio-Techne, GLR004, Minneapolis, MN, USA) was first reconstituted in 6.67 mL PBS to achieve a concentration of 15 mg/mL. The collagen hydrogel was formed as previously described but with the addition of 25 μL hyaluronic acid. All collagen hydrogels were allowed to set for 1 h in an incubator at 37°C before 100 μL of media was added on top of the hydrogel. The spheroids were first imaged after the media had been added and then every 24 h for 72 h using a Nikon TE2000 inverted microscope (Tokyo, Japan). The area of the spheroids after they had first been encased in the collagen was measured using ImageJ software (Bethesda, MD, USA) [47]. This is known as the spheroid core. The coefficient of variation of the diameter of the spheroids was then calculated as outlined in the protocol by Roper and Coyle [48]. If the coefficient of variation was less than 20% then the spheroid diameters were consistent between the spheroids and the results between spheroids were comparable. ImageJ was also used to measure the final area of the spheroid after the 72 h of invasion. The invasion area was calculated by taking the core area of the spheroid away from the final invasion area.

### 2.10 Statistical Analysis

All statistical analysis was carried out using GraphPad Prism 10 software (Boston, MA, USA). The normality of the data was first checked using a Shapiro-Wilk test. Data is presented as mean ± standard error. For data that was normally distributed and required the comparison of more than two mean values, a Brown-Forsythe and Welch analysis of variance (ANOVA) with Dunnet’s T3 multiple comparison post-hoc test was used as the samples had different standard deviations. For data that was normally distributed and required the comparison of just two mean values, an unpaired *t*-test with Welch’s correction was used as the samples had different standard deviations. Survival curves in Kaplan-Meier plots were compared using a log-rank test and multivariate analysis using Cox regression was used to evaluate the prognostic value of CD44 expression. *p*-values were considered to be significant if *p* < 0.05. The experimental repetitions are as follows, n = 49 for the staining of tissue sections, n = 3 for qRT-PCR, n = 5 for CCK-8 and n = 9 for the spheroid experiments.

## 3 Results

**Figure 1:**
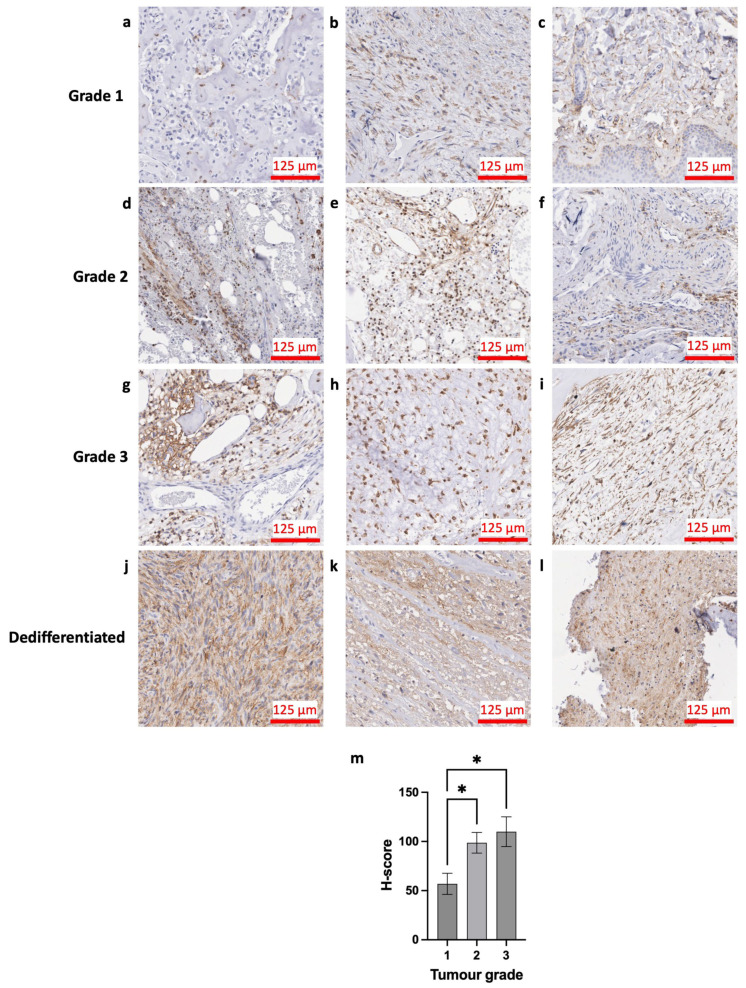
Staining of chondrosarcoma patient tissue sections. (**a**–**l**): Images of chondrosarcoma patient tissue that were stained with an antibody for Cluster of differentiation 44 (CD44) at a dilution of 1:500. Hematoxylin was used as a counterstain which stains cell nuclei purple. Images were scanned with a Leica Aperio CS2 at 20× magnification. Tissue sections from patients with different grades and types of chondrosarcoma were investigated: (**a**) clear cell, (**b**) atypical cartilaginous, (**c**–**f**), (**h**) and (**i**) conventional, (**g**) mesenchymal and (**j**–**l**) dedifferentiated. (**m**) The histochemical score (H-score) of the chondrosarcoma patient tissue slices that were stained with an antibody for CD44 was determined using the positive cell detection tool on QuPath software. The H-score values were grouped by tumour grade where n = 14 for grade 1, n = 17 for grade 2, n = 18 for grade 3. Statistical analysis was performed using the Brown-Forsythe and Welch ANOVA test with Dunnett’s T3 multiple-comparison post-hoc test. *p < 0.05.

To understand the expression of CD44 for *in vivo* chondrosarcoma tumours, immunohistochemistry was used to stain 49 chondrosarcoma tumour samples. Immunohistochemistry staining of the patient tissue showed that CD44 was expressed for all tumour grades (Fig. 1a–l). Positive CD44 expression was mainly observed in the cell membrane with some staining also in the cytoplasm. CD44 was generally found in clusters within the tissues except for the dedifferentiated chondrosarcoma samples where CD44 was more widespread. Quantification of the CD44 staining across the tissue samples using the H-score (Fig. 1m) showed that the staining intensity of the low grade (grade 1) tumours was significantly lower than the staining intensity of the intermediate grade (grade 2) and high grade (grade 3) tumours.

**Figure 2:**
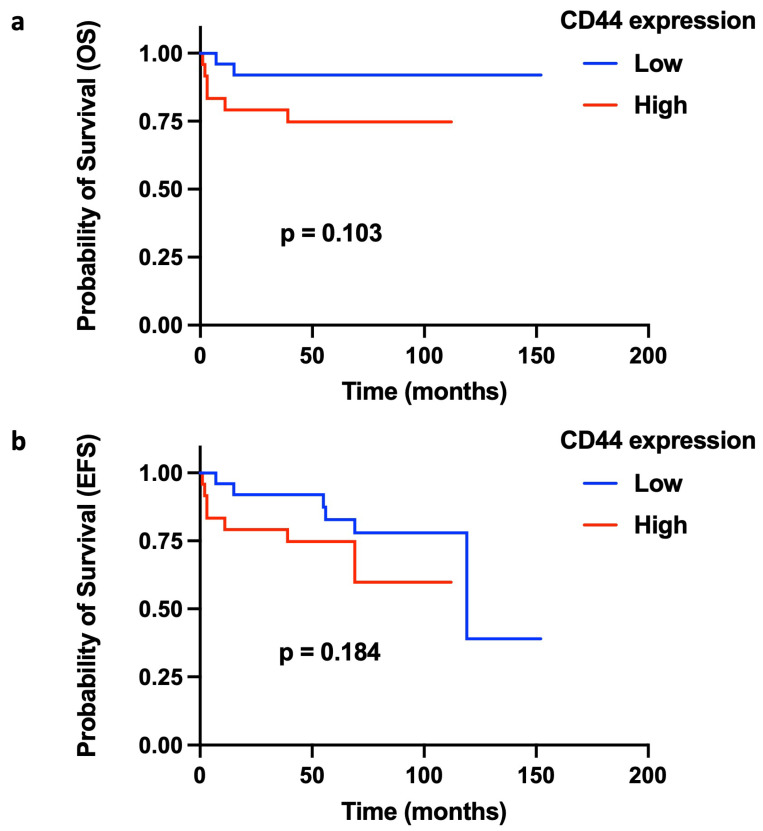
Survival analysis of Cluster of differentiation 44 (CD44) expression. (**a**) Kaplan-Meier plot of probability of overall survival (OS) against time for high (H-score > 84) and low (H-score < 84) CD44 expression. (**b**) Kaplan-Meier plot of probability of event-free survival (EFS) against time for high (H-score > 84) and low (H-score < 84) CD44 expression (n = 49).

To determine risk factors that may affect the OS and event free survival (EFS) of chondrosarcoma patients, survival analysis was undertaken. Kaplan-Meier survival analysis of CD44 expression suggested that high CD44 expression (H-score > 84) was not linked to decreased OS (Fig. 2a) or EFS (Fig. 2b). Multivariate analysis using Cox regression with tumour size, CD44 expression, presence of metastasis, age and sex was also undertaken (Table 2). Cox regression revealed that tumour size, age and sex did not lead to decreased OS or EFS. However, CD44 expression had a slight impact on OS (HR = 1.031, *p* = 0.0060) and EFS (HR = 1.020, *p* = 0.0057). Presence of metastasis also had an impact on OS (HR = 17.41, *p* = 0.0101) and EFS (HR = 13.35, *p* = 0.0002).

**Table 2:** This table contains the hazard ratio (HR) and *p*-values for overall survival (OS) and event-free survival (EFS) obtained from multivariate analysis calculated using Cox regression. A higher HR value corresponds to an increased risk. Bold *p*-values indicate that it was statistically significant (*p* < 0.05).

	OS		EFS	
	HR	*p*-Value	HR	*p*-Value
Tumor size	1.001	0.9184	1.002	0.4617
CD44 expression	1.031	**0.0060**	1.020	**0.0057**
Metastasis	17.41	**0.0101**	13.35	**0.0002**
Age	1.052	0.1234	1.044	0.1290
Sex	0.6488	0.7453	0.2177	0.1725

**Figure 3:**
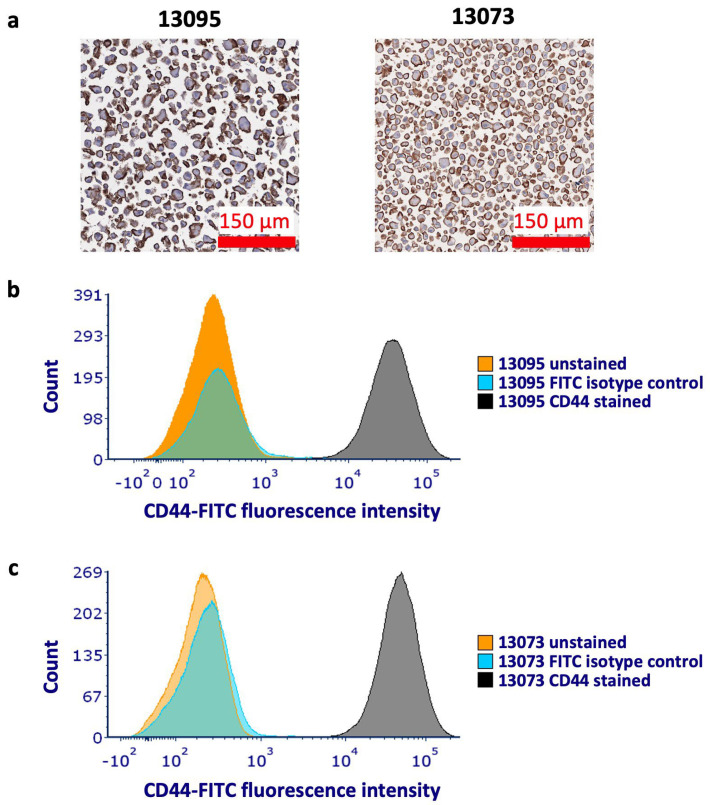
Characterisation of patient-derived chondrosarcoma cells. (**a**) Images of cell blocks of 13095 and 13073 patient-derived cells that were sliced and immunostained with an antibody for cluster of differentiation 44 (CD44) at a dilution of 1:1000. Brown staining indicates CD44 positive staining due to the oxidation of DAB. Hematoxylin was used as a counterstain which stains cell nuclei purple. Images were scanned with a Leica Aperio CS2 at 20× magnification. Flow cytometry was also used to analyse the surface expression of CD44 in (**b**) 13095 and (**c**) 13073 patient-derived chondrosarcoma cells. For both 13095 and 13073 cells, the cells were either left unstained, stained with a CD44 antibody conjugated to a fluorescein isothiocyanate (FITC) fluorophore or stained with a FITC isotype control. The *x*-axis of the histogram represents the fluorescence intensity (biexponential scale) and the *y*-axis represents cell count. Flow cytometry data was analysed using FCS Express 7 software.

To further understand CD44 expression in chondrosarcoma cells, patient derived chondrosarcoma cells were successfully cultured and embedded to paraffin blocks. The cell blocks were sliced and immunostained for CD44 (Fig. 3a). As with the patient tissue samples, the patient-derived cells showed strong positive staining for CD44, mainly in the cell membrane. Flow cytometry was used to further characterise the patient-derived cells for CD44 expression (Fig. 3b,c). Both the 13095 and 13073 cells strongly expressed CD44.

**Figure 4:**
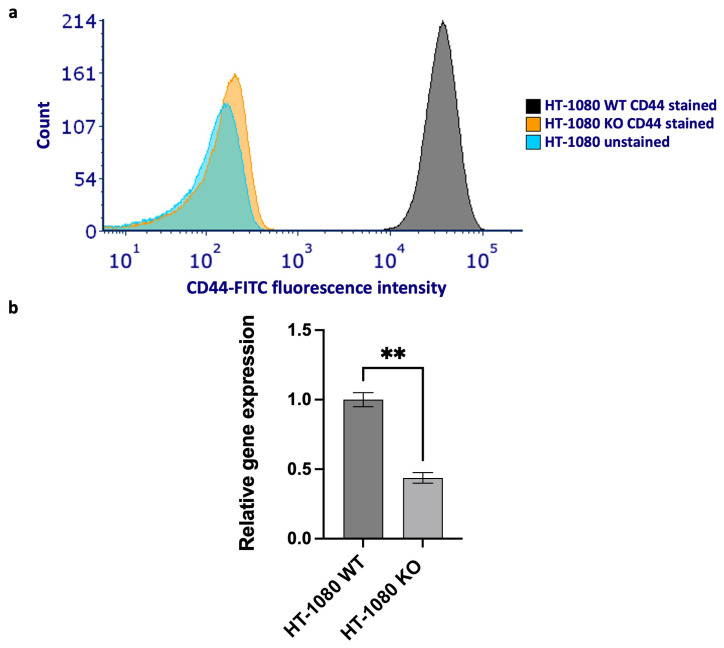
Characterisation of HT-1080 wild type (WT) and HT-1080 cluster of differentiation 44 (CD44) knockout (KO) cells. (**a**) Flow cytometry was used to analyse the surface expression of CD44 in HT-1080 WT and KO cells. The cells were either left unstained or stained with a CD44 antibody conjugated to a fluorescein isothiocyanate (FITC) fluorophore. The *x*-axis of the histogram represents the fluorescence intensity (biexponential scale) and the *y*-axis is the number of cells. There is a high intensity peak for the WT cells with lower intensity peaks for unstained and KO cells. Flow cytometry data was analysed using FCS Express 7 software. (**b**) Quantitative reverse transcription polymerase chain reaction (qRT-PCR) was used to investigate the CD44 mRNA levels in HT-1080 WT and CD44 KO cells. The relative gene expression was then calculated (n = 3, mean ± standard error of the mean). Statistical analysis was performed using an unpaired *t*-test with Welch’s correction. **p < 0.01 compared to the WT control.

**Figure 5:**
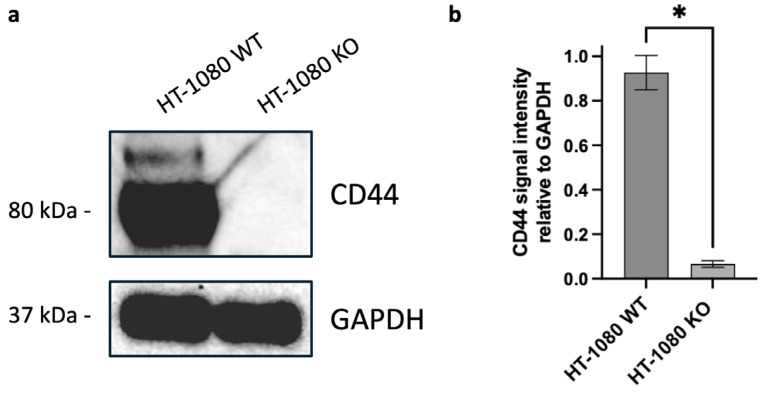
A western blot was used to investigate the protein level expression of cluster of differentiation (CD44) in the HT-1080 wild type (WT) and HT-1080 knockout (KO) cells. Glyceralydehyde-3-phosphate dehydrogenase (GAPDH) was used as the loading control. (**a**) The HT-1080 WT cells showed strong CD44 expression whilst the KO cells showed no significantly observable CD44 expression. (**b**) ImageJ was used to quantify the staining with respect to GAPDH (mean ± standard error of the mean). Statistical analysis was performed using an unpaired *t*-test with Welch’s correction. *p < 0.05 compared to the WT control.

**Figure 6:**
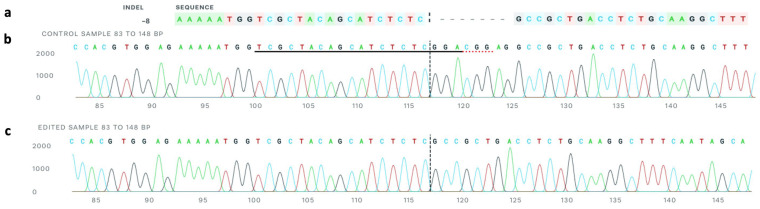
Sanger sequencing was performed on HT-1080 wild type (WT) and HT-1080 cluster of differentiation (CD44) knockout (KO) cell lines to compare their genetic sequences. (**a**) Shows the KO sequence where a negative indel value (−8) indicates that this clone contains just one sequence that has an 8 bp deletion compared to the WT sequence. The vertical black dashed line represents the cut site. (**b**) Shows the Sanger sequence of the WT (control sample) and (**c**) shows the Sanger sequence of the KO (edited sample). The guide sequence is underlined in black and the protospacer adjacent motif sequence (CGG) is underlined in a dashed red line on the control sample.

In order to determine how CD44 affects the invasion of chondrosarcoma cells, CRISPR/Cas9 gene editing was used to knockout CD44 from HT-1080 chondrosarcoma cells. Following FACS to separate out the KO cells into a single clone, flow cytometry was used to confirm that the knockout was successful at the cell surface expression level. The histogram of cell count against CD44 signal intensity (Fig. 4a) contained a low intensity peak for the unstained and CD44 stained sample as well as a high intensity peak for the stained WT sample. This suggests that at a surface level the CRISPR KO was successful. To investigate how the knockout affected CD44 messenger RNA (mRNA) expression levels in the KO cells, RNA was extracted from the WT and KO cells and qRT-PCR was used to measure the mRNA expression. The Ct values obtained from qRT-PCR were used to calculate the relative CD44 expression (Fig. 4b). There was a significant difference in gene expression between the WT cells and the CD44 KO cells (*p* < 0.01) however, the CD44 KO cells were still expressing some CD44. To understand CD44 expression at a protein level, a western blot was undertaken (Fig. 5). The missing band in the KO lane suggests that the KO was successful. To understand the genetic modifications that took place after the CRISPR transfection, Sanger sequencing was undertaken. The sequencing data was then analysed with Synthego ICE analysis which is a computational method that provides a summary of the composition of the sequences in the sample (Fig. 6). The results showed that the KO cells contained just one sequence that had a deletion of 8 bps compared to the WT sequence and the deletion occurred at the cut site of the cas9 nuclease enzyme.

**Figure 7:**
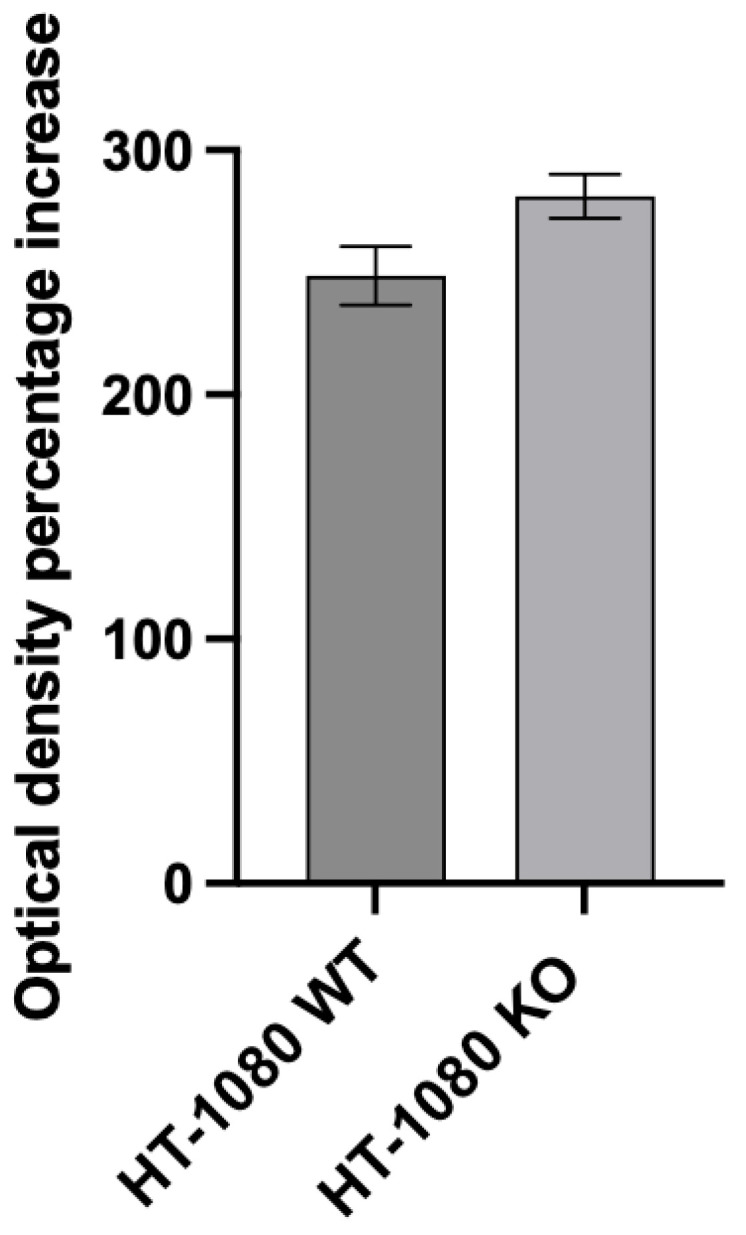
A cell counting kit-8 (CCK-8) assay was used to compare the proliferation of the HT-1080 wild type (WT) and HT-1080 cluster of differentiation 44 (CD44) knockout (KO) cells. The proliferation rate was determined by the optical density percentage increase after 72 h incubation period, compared to day 1 for both the WT and KO cell lines. The results of this analysis are shown in the bar chart (mean ± standard error of the mean).

A CCK-8 cell proliferation assay was then used to compare the proliferation rates of the HT-1080 WT and HT-1080 CD44 KO cells. Cells were seeded at the same density and then incubated for 72 h, the same time period as the invasion experiments. After 1 day, the optical density was measured and then re-measured at the 72 h point. The percentage increase in the optical density between these two time points was calculated as a measure of proliferation rate (Fig. 7). The mean percentage increase for the WT and KO cell lines was 248.6 ± 33.3 and 281.1 ± 25.0 respectively. There was no significant difference between the percentage increase of the optical densities of the WT and KO cells after 72 h following analysis by Welch’s *t*-test (*p* = 0.0649). Although not statistically significant, the observed mean percentage increase was in fact higher for the HT-1080 KO cell line, which suggests that CRISPR KO of CD44 did not negatively impact the proliferation rate of the KO cell line which may have confounded the results of the invasion assays.

**Figure 8:**
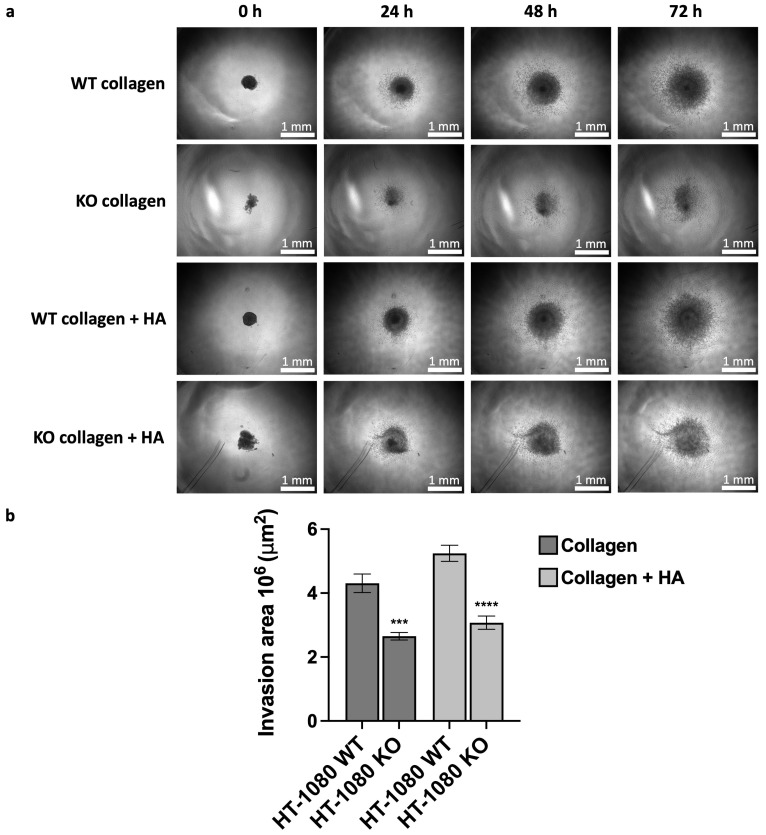
HT-1080 wild type (WT) vs. cluster of differentiation 44 (CD44) knockout (KO) collagen spheroid invasion assay. HT-1080 WT and CD44 KO spheroids were encased in collagen or collagen with hyaluronic acid (HA) added for 72 h. (**a**) The spheroids were imaged every 24 h. Images were taken using a Nikon Eclipse TE2000 inverted microscope at 2× magnification. (**b**) Quantification of the spheroid invasion. ImageJ software was used to measure the initial and final spheroid areas and the invasion area was calculated as final invasion area after 72 h—initial area of the core. Each cell type and hydrogel composition were tested in triplicate and each experiment was repeated three times (n = 9, mean ± standard error of the mean). Statistical analysis was performed using an unpaired *t*-test with Welch’s correction. ***p < 0.001, ****p < 0.0001 compared to the WT cells in the same hydrogel composition.

To investigate the effects of the CD44 knockout on the invasion of the HT-1080 cells, WT and CD44 KO spheroids were encased in a collagen hydrogel or a collagen hydrogel with hyaluronic acid added and allowed to invade for 72 h. The spheroid invasion was imaged every 24 h (Fig. 8a) and the final invasion area of the spheroids after the 72 h was calculated (Fig. 8b). When the spheroids were encased in just collagen, there was a significant decrease (*p* < 0.001) in the invasion of the KO cells compared to WT. There was also a decreased invasion (*p* < 0.0001) for the KO cells compared to the WT cells for the spheroids encased in collagen with hyaluronic acid.

## 4 Discussion

The aim of this study was to investigate the expression of CD44 in chondrosarcoma, and further understand its role in disease progression. This work represents the largest current study to date on CD44 expression in chondrosarcoma patient tissue. Through immunohistochemical staining, it was shown that all chondrosarcoma tissue samples from 49 different tissue sections expressed some level of CD44. This confirms that CD44 is ubiquitously expressed in chondrosarcoma tissue *in vivo* and highlights that CD44 could be a possible future therapeutic or diagnostic target for chondrosarcoma. Quantification of the CD44 positive staining revealed significantly increased CD44 expression in the grade 3 and grade 2 tissue sections when compared to grade 1. Grade 3 and dedifferentiated chondrosarcomas have the highest risk of metastasis [49]. This suggests that the increased invasion and metastasis of high-grade chondrosarcomas could be due to increased CD44 expression. In a study by Xu et al., breast cancer tissue microarray slides were stained for CD44 and the CD44 expression increased with tumour grade [50]. This study did not find a significant difference between CD44 expression between intermediate and high grades, suggesting that even for grade 2 tumours CD44 expression is high and could be an important marker.

Kaplan-Meier analysis was undertaken to understand whether CD44 expression influenced OS and EFS. The results showed that CD44 expression did not have an effect on the survival and outcomes in our studied cohort. In a previous study by Heyse et al. where CD44 expression in tissue from 30 chondrosarcoma patients was investigated, Kaplan-Meier analysis revealed that CD44s did have an effect on both OS and metastatic free survival but CD44v5 did not [34]. In this study, the CD44 antibody used was not isoform specific therefore, the lack of significance could be due to detecting cells which are expressing a variant form of CD44 rather than the standard form which could have an increased effect on patient prognosis. However, it has been shown that for some chondrosarcoma subtypes CD44v is more highly expressed than standard isoforms of CD44 [51]. Therefore, the use of a pan-CD44 antibody still provides an insight into chondrosarcoma because both standard and variant isoforms of CD44 have been shown to be overexpressed in invasive chondrosarcoma cells. A pan-CD44 antibody has been used to investigate CD44 expression across tissue samples from 53 osteosarcoma patients. Kaplan-Meier analysis highlighted a shorter OS in patients expressing CD44 compared to patient samples with undetectable staining [52]. A similar result to the one observed in this study was also obtained by Neumeister et al. where Kaplan-Meier analysis showed that CD44 expression was not linked to survival in a breast cancer cohort [53]. Therefore, further studies need to be undertaken with a larger number of samples to understand if CD44 expression can predict patient prognosis.

Cox regression, however, did suggest that CD44 expression can marginally contribute to patient prognosis as the results of the regression were significant but the HR values were only slightly elevated. This could imply that CD44 expression alone cannot predict the prognosis of a patient but combined with other factors, it could contribute to reduced OS and EFS. Multivariate analysis of progression-free survival and CD44 expression in non-small-cell lung cancer tumours also showed that CD44 expression did affect the progression-free survival [54]. In a similar study where 44 myxofibrosarcoma tissue samples were stained for CD44s, the expression of CD44s in the tissues was also not able to predict patient prognosis but multivariate analysis did show that high expression of CD44s contributed to worse EFS [55]. The largest HR values that were obtained from the multivariate analysis were for metastasis which shows that metastasis could result in a poor prognosis for chondrosarcoma patients. This is consistent with results obtained by Brown et al. where a study on 472 patients with chondrosarcoma of the pelvis found that patients with metastatic chondrosarcoma had a 5-year survival of 14.7% but the patients without metastasis had a 5-year survival rate of 67.4% [56]. This highlights the importance of investigating factors that can affect tumour cell invasion because if invasion is prevented or reduced then metastasis might also be reduced. Although this study did not directly correlate CD44 expression with oncological outcome, the high prevalence of CD44 expression by the chondrosarcoma cells could still be important for the development of immunotherapies such as antibody-drug conjugates. For example, an effective tetravalent agonist that binds to death receptor 5 has been explored for the treatment of relapsed chondrosarcoma which does not include expression levels of the target [57].

Patient-derived chondrosarcoma cells from two patients were also successfully cultured and characterised for CD44 expression for the first time in this work. The successful culture and characterisation of these cells is promising for the development of patient-specific treatments. Strong CD44 expression was measured in both the grade 2 and grade 3 patient-derived cells which is important as even intermediate grades of chondrosarcoma have a high metastatic potential [58]. The ability to successfully culture patient-derived cells is important because it may allow for the development of personalised treatments [59].

Finally, for the first time to our knowledge, CRISPR/Cas9 gene editing was used to knockout CD44 in the HT-1080 dedifferentiated chondrosarcoma cell line. CRISPR was used to knock out CD44 from the cell lines as compared to a knockdown where a gene is silenced; a genetic knockout is precise, often has fewer off-target effects and, is permanent within the KO clone [60]. Results obtained from flow cytometry and western blot suggest that, CD44 was no longer expressed by the KO cells but qRT-PCR results suggest that although significantly less than the WT cells, the KO cells still express some CD44 mRNA. In previous work by Kapahnke et al. CRISPR was used to knockout the flotillin-1 gene from HeLa cells [61]. Similarly to this study, qRT-PCR showed that some of the HeLa KO cells had similar mRNA expression levels as the WT cells. mRNA sequencing of the clones showed that for a KO clone that still had high levels of mRNA expression, an exon had been deleted and alternative splicing had occurred. However, in this study Sanger sequencing showed that at a DNA level, the knockout was a full homogenous knockout.

A 3D spheroid invasion assay was then used to investigate the effect of the knockout on chondrosarcoma cell invasion for the first time in this work. Spheroid invasion assays are a useful technique that can represent the *in vivo* tumour and cell-cell interactions [62]. The spheroid invasion assay showed that the CD44 KO cells invaded significantly less than the WT cells. This suggests that CD44 does play an important role in chondrosarcoma cell invasion and targeting CD44 could reduce further invasion and metastasis for chondrosarcoma tumours. The results of the CCK-8 proliferation assay showed that there was no statistically significant difference between the proliferation rate of the HT-1080 WT and KO cells over 72 h. The observed mean percentage increase was in fact higher for the KO cell line, therefore the CRISPR CD44 KO did not negatively influence the cell proliferation rate and is unlikely to have confounded the results of the invasion assays. Furthermore, even though there was CD44 mRNA detected in the KO cells which may have resulted in functional protein isoforms, the reduction in invasion suggests that the isoforms may not have been beneficial. The 8 bp deletion in the knockout cells detected by the Sanger sequencing was therefore likely to have disrupted the function of the CD44 gene. The role of CD44 in the invasion of Ewing sarcoma cells has previously been investigated. Spheroids of Ewing sarcoma cells from three Ewing sarcoma cell lines were encased in a collagen matrix and treated with doxycycline which can downregulate CD44 expression. Spheroids generated from two of the cell lines which were not treated with doxycycline showed increased invasion compared to the treated spheroids in accordance to the results of this study [63].

Hyaluronic acid was also added to the collagen hydrogel surrounding the spheroids because it is a key component of the ECM and the main ligand for CD44 [64]. There was a significant increase in invasion for the HT-1080 WT spheroids encased in the collagen hydrogel with hyaluronic acid compared to just the collagen hydrogel. Furthermore, the difference between the invasion area for the WT and CD44 KO cells was greater for the spheroids that were encased in the hydrogel containing hyaluronic acid. When CD44 interacts with hyaluronic acid, several proteins are activated including RhoA which leads to reformation of the actin cytoskeleton, allowing cells to migrate. Hyaluronic acid binding to CD44 also activates MMP-9 which can degrade collagen, allowing tumour cells to invade [65]. Hyaluronic acid can also reduce cell attachment to the ECM which allows for easier cell migration [66]. Therefore, the presence of hyaluronic acid as well as CD44 could be an important factor for chondrosarcoma cell invasion. The spheroid model could be developed further to make the system more representative of the *in vivo* system. For example, a co-culture spheroid could be used with the inclusion of monocytes which have been shown to be recruited by chondrosarcoma cells [67].

The HT-1080 cells that were used in this study harbour an IDH1 mutation and the presence of IDH1 and IDH2 mutations are common for chondrosarcoma cells [68]. Previously, Lyu et al. identified that CD44 was activated by leukaemia cells harbouring IDH mutations [69]. However, glioma patients with IDH mutated cells have shown low levels of CD44 expression [70]. Therefore, future studies could investigate how the presence of IDH mutations in chondrosarcoma affect CD44 expression and disease progression.

The promising results of this study suggest that targeting CD44 could be a beneficial treatment option for chondrosarcoma patients although further validation with larger cohorts and *in vivo* models are needed. The reduction of CD44 in patients could be achieved at a transcriptional level using gene therapy. Previously, a CD44 short hairpin RNA lentiviral vector was used to down-regulate CD44 in breast cancer tumour bearing mice. Tumour size was decreased in the mice treated with gene therapy compared to the control [71]. Gene therapy can also be achieved using small interfering RNA to knockdown CD44. Human colon cancer xenografts exposed to CD44 siRNA plasmids exhibited reduced tumour volume [72]. Recently, lipid nanoparticles have also successfully been used to target CD44 on melanoma cells to deliver CRISPR/Cas9 gene editing which was able to reduce tumour growth *in vitro* and *in vivo* [73]. CD44 levels can indirectly be altered using some tyrosine kinase inhibitors such as erlotinib which blocks epidermal growth factor activity. Erlotinib was shown to reduce CD44 expression by squamous cell carcinoma cells [74]. Overexpression of CD44 by cancer cells may allow for targeted treatments to be delivered to tumour cells resulting in reduced treatment toxicity to the patient. For example, nanoparticles containing the chemotherapy agent paclitaxel were coated with hyaluronic acid and used to treat mice in a breast cancer xenograft model. Coating the nanoparticles in hyaluronic acid resulted in increased treatment uptake and therefore reduced tumour volume [75].

A limitation of this study is the observed differences in the contribution of increased CD44 expression to patient survival shown by the Kaplan-Meier survival analysis and Cox multivariate analysis. As previously discussed, the lack of significance shown in the Kaplan-Meier analysis could be due to the use of a non-isoform specific CD44 antibody which would need to be addressed for future studies to fully understand the impact of CD44 expression on patient survival. Furthermore, although this study is the largest study of its kind to date, the study could be expanded to increase the patient cohort and to include a larger number of secondary tumour samples to investigate CD44 expression of primary against secondary tumour samples. Addressing these limitations in future studies would allow for a greater understanding of the role of CD44 in chondrosarcoma. Clinically, this could mean that CD44 expression could be used to predict patient outcomes, or CD44 could be a potential therapeutic target for the treatment of chondrosarcoma.

## 5 Conclusions

The results from this study highlight that CD44 may be an important factor in the invasion and progression of chondrosarcoma. However, the varying results obtained from survival analysis highlight the need for future studies, with the use of isoform specific antibodies and larger patient cohorts to understand whether CD44 expression could be an independent predicator of chondrosarcoma prognosis. The clinical trial RG7356 and its subsequent imaging studies [32,33] combined with the data obtained in this study, suggest that CD44 could be a useful imaging and therapeutic target for chondrosarcoma. Future work should focus on substantiating these results using *in vivo* models, to better determine the translational applicability of CD44 in the management of chondrosarcoma.

## Data Availability

The data that supports the findings of this study are available from the corresponding author, [Kenneth S. Rankin], upon reasonable request.

## Abbreviations

The following abbreviations are used in this manuscript:

ANOVA	Analysis of variance
Bp	Base pair
BSA	Bovine serum albumin
Cas9	CRISPR associated protein 9
CD44	Cluster of differentiation 44
cDNA	Complementary deoxyribonucleic acid
CRISPR	Clustered regularly interspaced short palindromic repeats
DAB	3,3′-diaminobenzidine
DI	De-ionised
ECM	Extracellular matrix
EFS	Event-free survival
EMT	Epithelial-mesenchymal transition
FACS	Fluorescence activated cell sorting
FITC	Fluorescein isothiocyanate
GAPDH	Glyceraldehyde-3-phosphate dehydrogenase
H-score	Histochemical score
HR	Hazard ratio
IDH	Isocitrate dehydrogenase
KO	Knockout
mRNA	Messenger RNA
MMP-9	Matrix metalloproteinase-9
OS	Overall survival
PCR	Polymerase chain reaction
PET	Positron Emission Tomography
PBS	Phosphate-buffered saline
qRT-PCR	Quantitative reverse transcription PCRpolymerase chain reaction
RhoA	Ras homolog family member A
ROCK	Rho-associated coiled coil containing protein kinase
RPMI	Roswell Park Memorial Institute
sgRNA	Single guide ribonucleic acid
TBS	Tris-buffered saline
ULA	Ultra-low attachment
WT	Wild type
